# Hyperuricemia is associated with metabolic syndrome in the community very elderly in Chengdu

**DOI:** 10.1038/s41598-020-65605-w

**Published:** 2020-05-26

**Authors:** Gang Huang, Junbo Xu, Tingjie Zhang, Lin Cai, Hanxiong Liu, Xiuqiong Yu, Jing Wu

**Affiliations:** 1 Cardiovascular Disease Research Institute of Chengdu, Sichuan, China; 2 Department of Cardiovascular Disease, The Third People’s Hospital of Chengdu, Chengdu, China; 3 Affiliated Hospital of Southwest Jiaotong University, Sichuan, China; 4 The Second Affiliated Chengdu Clinical College of Chongqing Medical University, Sichuan, China

**Keywords:** Cardiovascular diseases, Metabolic syndrome

## Abstract

Hyperuricemia is a risk factor for cardiovascular metabolic diseases. However, in the very elderly, the relationship between hyperuricemia and the metabolic syndrome (MetS) is not yet clear. This study was aimed to investigate the potential association between hyperuricemia and MetS in community very elderly in Chengdu. In this cross-sectional study, 1056 very elderly in the community were enrolled. Serum uric acid (SUA), fast plasma glucose, triglycerides and high–density lipoprotein cholesterol were measured, and then MetS components were calculated. Logistic regression models were used to explore risk factors for MetS in the very elderly. Finally, 1035 participants were included in analysis whose ages ranged between 80 and 100 with a mean age of 83.6 ± 3.4 years. The mean SUA level was 356.2 ± 95.0 µmol/L. The estimated prevalence of MetS in the very elderly was 25.0% vs. 21.6% (international diabetes federation (IDF) criteria vs. Chinese guideline), which was significantly higher for women (IDF criteria:17.3% in men vs 33.6% in women, p < 0.001). Logistic regression has found that participants with hyperuricemia (SUA level > 416 µmol/L in men and > 357 µmol/L in women) had a higher risk (IDF criteria: odds ratio (OR): 2.136, 95% confidence interval(CI): 1.525–2.993, p < 0.001. Chinese guideline: OR: 1.769, 95%CI: 1.249–2.503, p = 0.001) of MetS in very elderly Chinese. MetS is common in the community of very elderly Chinese in Chengdu. Hyperuricemia is associated with MetS in general very elderly and lifestyle changing should also be considered in the very elderly.

## Introduction

As a worldwide epidemic clinical syndrome nowadays, metabolic syndrome (MetS) is characterized by co-occurrence of abdominal obesity, hyperglycemia, dyslipidemia, and hypertension. It has been shown that MetS is a well-known risk factor for Type 2 diabetes mellitus (DM), and atherosclerotic cardiovascular disease (ASCVD) morbidity and mortality, resulting in an enormous economic burden to society^1^. A national survey has reported that the prevalence of MetS in Chinese aged from 35 to 74 years was about 10% and 18% in men and women, respectively, in 2000–2001^2^. However, the prevalence of MetS is increasing with social-economic growth and undergoing lifestyle transition. Another recent national survey has demonstrated that the prevalence of MetS in Chinese older than 18 years was 31.0% in men and 36.8% in women^3^. Meanwhile, China has become an aging society with a proportion of elderly of about 1.6% in 2010 accompanying with great economic achievements. Since ASCVD has already become the first disease burden in China^4^, MetS with an increasing prevalence has thus become a big public health challenge to prevent ASCVD in China.

Serum uric acid (SUA) is the end product of purine metabolism in humans, which has been reported as a risk factor for hypertension^5,6^, atrial fibrillation^7,8^, acute myocardial infarction^9^, ischemic heart disease^10^, and stroke^11^. Although numerous studies^12–15^ have demonstrated the association between SUA level and MetS in young, middle-aged and elderly population, little is known about the association between them in the very elderly. Moreover, this group has not usually been included in epidemiological or clinical studies, even less as a unique study group. Therefore, this study aimed to investigate the potential association between hyperuricemia and MetS in the very elderly in Southwest China.

## Methods

### Subjects

This cross-sectional study was designed to detect the cardiovascular and metabolic risk factors in the general community of the very elderly population (≥80 years old) in Chengdu, which was described elsewhere^16^. From 2013 to 2015, a representative sample of the community very elderly was recruited using of a stratified three-stage cluster sampling design. A total of 1056 very elderly from 20 residential communities were taken according to the registration data from local government^16^. The study protocol conforms to the ethical guidelines of the 1975 Declaration of Helsinki as reflected in a prior approval by the Ethics Committee of the Second People’s Hospital of Chengdu. Moreover, all participants gave their informed consent.

### Data collection and measurement

Previously well-trained physicians and nurses collected data from the participants with a questionnaire-based face to face interview during clinical visits, which was described elsewhere^16^. Basic demographic characteristics, past medical history and lifestyle risk factors of participants were obtained from a standardized questionnaire during the interview with participating investigators. The body mass index (BMI) was defined as weight in kilograms divided by the square of the height in meters. The blood pressure (BP) was measured three times in a sitting position by using a standardized automatic electronic sphygmomanometer (HEM-7300, Omron, Kyoto, Japan) and average values were calculated and used in the analysis. Fasting blood samples were collected in the morning after at least 8 hours of fasting for all participants. SUA and other biochemical parameters, such as fast plasma glucose(FPG), total cholesterol(TC), triglycerides(TG), and low–density lipoprotein cholesterol(LDL-C) were analyzed enzymatically on an auto-analyzer (AU5421 Chemistry Analyzer, Beckman, Brea, California, United States) in the central laboratory.

Finally, the estimated glomerular filtration rate (eGFR) was calculated using the Modification of Diet in Renal Disease study equation modified for the Chinese population: eGFR = 186 × serum creatinine^−1.154^ × Age^−0.203^ × 0.742 (if female).

### Definitions

hyperuricemia was defined as SUA level >416 µmol/L (7.0 mg/dL) in men and >357 µmol/L (6.0 mg/dl) in women^17^.

MetS, according to the Consensus Worldwide Definition from the international diabetes federation (IDF)^18^ and the Chinese guideline for dyslipidemia management^19^, was defined as follows, respectively:

MetS defined by IDF: abdominal obesity with ethnic-specific waist circumference (WC) cut-points (for Chinese: ≥ 90 cm in men and ≥ 80 cm in women) and fulfills two items of the following: TG ≥ 150 mg/dL (1.7 mmol/L) or treatment for hypertriglycerides, HDL cholesterol <40 mg/dL (1.03 mmol/L) in men or <50 mg/dL (1.29 mmol/L) in women or treatment for low HDL, FPG ≥ 100 mg/dL (5.6 mmol/L) or previously diagnosed type 2 diabetes, and BP ≥ 130/85 mmHg or treatment for hypertension.

MetS defined in the Chinese guideline: MetS should fulfill any three or more of the following criteria: abdominal obesity (WC ≥ 90 cm in men and ≥85 cm in women), fasting TG ≥ 150 mg/dL (1.7 mmol/L), fasting HDL-C < 40 mg/dL (1.0 mmol/L), FPG ≥ 110 mg/dL (6.10 mmol/L) or a 2 hour blood glucose after glycemic load ≥140 mg/dL (7.80 mmol/L) or anti-diabetic treatment, and BP ≥ 130/85 mmHg or anti-hypertensive treatment.

### Statistical analysis

SPSS software (Version 22.0, SPSS Inc, Chicago, IL) were used to performed the statistical analyses. Continuous variables with normal distribution are expressed as mean ± standard deviation and skewed continuous variables expressed as median (interquartile range). Frequencies are presented as percentages with 95% confidence interval (95%CI). ANOVA or Kruskal-Wallis test was used to compare the continuous variables between groups, whereas x^2^ test was applied to compare frequencies. Logistic regression models were used to evaluate the potential association between hyperuricemia and MetS. The receiver operating characteristic (ROC) curve analysis was employed to evaluate the efficiency of SUA level in predicting MetS. A two-sided *p* value < 0.05 was considered statistically significant.

## Results

### Baseline characteristics

A total of 1035 participants with a mean age of 83.6 ± 3.4 years were enrolled in the final statistical analysis, of which 52.6% were men with a mean age of 83.6 ± 3.3 years. The overall mean SUA level was 356.2 ± 95.0 µmol/L with a mean level of 374.2 ± 94.5 µmol/L in men and 335.2 ± 89.3 µmol/L in women (p < 0.001). According to both sets of criteria explained above, participants with MetS had a significantly higher mean SUA level (IDF criteria: 375.8 ± 90.8 vs. 349.5 ± 95.6 µmol/L, p < 0.001; Chinese guideline: 375.2 ± 88.8 vs. 350.7 ± 96.0 µmol/L, p < 0.001). The overall mean level of eGFR was 58.4 ± 13.9 ml/(min∙1.73m^2^) (men vs. women: 61.8 ± 14.1 vs. 54.6 ± 12.6 ml/(min∙1.73m^2^), p < 0.001). As shown in Table 1, participants with more MetS components were more likely to be abdominal obese with higher levels of WC, BMI, SBP, DBP, FBG, TC, TG, LDL and SUA, but a lower level of HDL.

**Table 1:** Characteristics of community very elderly Chinese in Chengdu.

	Number of MetS components				p value
	0 (n = 72)	1 (n = 328)	2 (n = 348)	≥ 3 (n = 287)	
Age (yrs)	83.9 ± 3.3	83.7 ± 3.5	83.6 ± 3.4	83.6 ± 3.4	0.551
Current smoker, n(%)	7(9.7)	42(12.8)	34(9.8)	31(10.8)	0.624
Current drinker, n(%)	4(5.6)	27(8.2)	30(8.6)	25(8.7)	0.844
**Medical history, n(%)**					
Hypertension	16(22.2)	152(46.3)	200(57.5)	178(62.0)	<0.001
DM	2(2.8)	28(8.5)	59(17.0)	88(30.7)	<0.001
Abdominal obesity	0(0)	46(14.0)	235(67.5)	257(89.5)	<0.001
**Medication, n(%)**					
Antihypertensive	11(15.3)	129(39.3)	170(48.9)	160(55.7)	<0.001
Antidiabetic	1(1.4)	18(5.5)	41(11.8)	70(24.4)	<0.001
Lipid lowering	1(1.4)	22(6.7)	32(9.2)	31(10.8)	0.040
Diuretics	1(1.4)	20(6.1)	26(7.5)	21(7.3)	0.237
WC (cm)	76.9 ± 6.9	80.0 ± 9.2	89.4 ± 9.0	94.1 ± 8.0	<0.001
BMI (kg/m^2^)	19.9 (17.5,21.9)	20.7 (18.9,23.1)	23.5 (21.6,25.9)	24.8 (22.9,27.3)	<0.001
SBP (mmHg)	120.0 (110.0,123.0)	142.0 (129.0,157.0)	148.0 (136.5,164.0)	151.5 (140.0,166.8)	<0.001
DBP (mmHg)	69.0 (62.0,72.5)	73.0 (64.0,81.0)	74.0 (67.0,82.3)	76.0 (68.0,83.0)	<0.001
FBG (mmol/L)	4.80 (4.42,5.24)	4.90 (4.50,5.27)	5.10 (4.67,5.80)	6.00 (5.22,7.56)	<0.001
TC (mmol/L)	4.64 (4.22,5.23)	4.77 (4.08,5.36)	4.94 (4.21,5.56)	4.88 (4.21,5.66)	0.018
TG (mmol/L)	1.04 (0.84,1.30)	0.99 (0.77,1.27)	1.17 (0.89,1.48)	1.76 (1.21,2.29)	<0.001
LDL (mmol/L)	2.42 (1.98,2.79)	2.44 (1.95,2.93)	2.57 (2.06,3.05)	2.69 (2.26,3.26)	<0.001
HDL (mmol/L)	1.67 (1.45,1.92)	1.65 (1.40,1.96)	1.59 (1.34,1.93)	1.27 (1.09,1.54)	<0.001
SUA (µmol/L)	324 (275.8,412.3)	334.5 (287.8,401.5)	344.0 (281.0,400.3)	373.0 (313.0,440.8)	<0.001
Creatinine (μmol/L)	98.0 (87.0,115.5)	97.5 (88.8,113.0)	98.0 (86.0,114.8)	97.5 (87.0,115.0)	0.225
e GFR, ml/(min∙1.73m^2^)	59.0 (43.0,73.2)	59.4 (51.0,69.3)	57.5 (48.9,64.9)	55.4 (46.1,65.0)	0.467

Data are expressed as mean ± standard deviation for normal distributed continuous variables, median (interquartile range) for skewed continuous variables, or number (percentage) for categorical variables. BMI: body mass index; DBP: diastolic blood pressure; DM: diabetes mellitus; eGFR: estimated glomerular filtration rate; HDL: high-density lipoprotein; LDL: low-density lipoprotein; SUA: serum uric acid; SBP: systolic blood pressure; TC: total cholesterol; TG: triglyceride; WC: Waist circumference.

### Estimated prevalence of MetS and its components

The overall estimated prevalence of MetS in the very elderly was 25.0% (17.3% in men vs. 33.6% in women, p < 0.001) according to the IDF criteria, which was significantly higher (p < 0.001) than that of the Chinese guideline (21.6%, 17.8% in men vs. 25.8% in women, p = 0.002). Table 2 describes the prevalence of its components.

**Table 2:** Estimated prevalences of metabolic syndrome and its components in community very elderly Chinese in Chengdu.

	IDF criteria			Chinese guideline		
	Men	Women	p value	Men	Women	p value
MetS (%)	95 (17.3)	165 (33.6)	<0.001	97 (17.8)	127 (25.8)	0.002
Abdominal obesity (%)	243 (44.5)	375 (76.2)	<0.001	243 (44.5)	307 (62.4)	<0.001
High blood pressure (%)	432 (79.1)	398 (80.9)	0.484	432 (79.1)	398 (80.9)	0.484
Hypertriglyceridemia (%)	106 (19.4)	127 (25.8)	0.013	106 (19.4)	127 (25.8)	0.013
Low HDL cholesterol (%)	49 (9.0)	106 (21.5)	<0.001	48 (8.8)	23 (4.7)	0.012
Hyperglycemia (%)	191 (34.9)	154 (31.3)	0.233	135 (24.7)	109 (22.2)	0.371

HDL: high-density lipoprotein,IDF: international diabetes federation, MetS: metabolic syndrome.

### Estimated prevalence of MetS in the very elderly with hyperuricemia

The estimated prevalence of MetS in participants with hyperuricemia was 39.4% vs. 32.9% (p < 0.001) according to the IDF and Chinese guideline, respectively. The estimated prevalence of MetS in very elderly women with hyperuricemia was significantly higher than it was in men according to both the IDF criteria (66.9% vs. 33.1%, p < 0.001) and the Chinese guideline (39.3% vs. 26.6%, p = 0.016).

### Relationship between hyperuricemia and MetS

After adjusting for sex, BMI, low-density lipoprotein, eGFR, cigarette smoking, total cholesterol, low-density lipoprotein cholesterol, alcohol consumption and medications for hypertension, DM, and hyperlipidemia, the logistic regression analysis of all cases (Table 3) demonstrated that hyperuricemia was an independent factor for MetS according to both the IDF criteria (odds ratio (OR): 2.136, 95%CI: 1.525–2.993, p < 0.001) and the Chinese guideline (OR: 1.769, 95%CI: 1.249–2.503, p = 0.001) in overall participants (Table 3). Moreover, the same finding was also found between SUA level and MetS for both the IDF criteria (OR: 1.342, 95%CI: 1.102–2.305, p < 0.001) and the Chinese guideline (OR: 1.215, 95%CI: 1.109–2.304, p < 0.001). Also, in subgroup analysis, hyperuricemia is associated with MetS both in very elderly women (OR: 2.324, 95%CI: 1.500–3.601, p < 0.001) and in men (OR: 1.920, 95%CI: 1.128–3.267, p = 0.016). It is noteworthy mentioning that both SUA level and hyperuricemia were also associated with two components of MetS, which were abdominal obesity and hypertriglyceridemia (Table 3).

**Table 3:** Association between hyperuricaemia and metabolic syndrome using logistic regression models in community very elderly Chinese in Chengdu.

	Serum uric acid OR (95% CI)	Hyperuricemia OR (95% CI)
Metabolic syndrome (IDF criteria)	1.342 (1.102–2.305)*	2.136 (1.525–2.993)*
**Metabolic syndrome component**		
Abdominal Obesity	1.320 (1.151–2.412)*	1.833 (1.204–2.792)*
High blood pressure	1.120 (0.898–1.503)	0.953 (0.615–1.477)
Hypertriglyceridemia	1.303 (1.122–2.205)*	1.843 (1.301–2.612)*
Low HDL cholesterol	1.352 (1.182–2.967)*	1.974 (1.292–3.017)*
Hyperglycemia	1.242 (1.156–1.881)*	1.330 (0.931–1.901)
Metabolic syndrome (Chinese guideline)	1.215 (1.109–2.304)*	1.769 (1.249–2.503)*
**Metabolic syndrome component**		
Abdominal Obesity	1.306 (1.138–2.326)*	1.550 (1.052–2.284)*
High blood pressure	1.120 (0.898–1.503)	0.953 (0.615–1.477)
Hypertriglyceridemia	1.303 (1.122–2.205)*	1.843 (1.301–2.612)*
Low HDL cholesterol	1.121 (0.797–1.304)	1.706 (0.566–2.046)
Hyperglycemia	1.102 (0.898–1.602)	1.114 (0.754–1.647)

Adjusted for sex, body mass index, low-density lipoprotein, eGFR, cigarette smoking, total cholesterol, low-density lipoprotein, alcohol consumption and medications for hypertension, diabetes mellitus, and hyperlipidemia. eGFR: estimated glomerular filtration rate. HDL: high-density lipoprotein, IDF: international diabetes federation. *p < 0.05.

**Figure 1:**
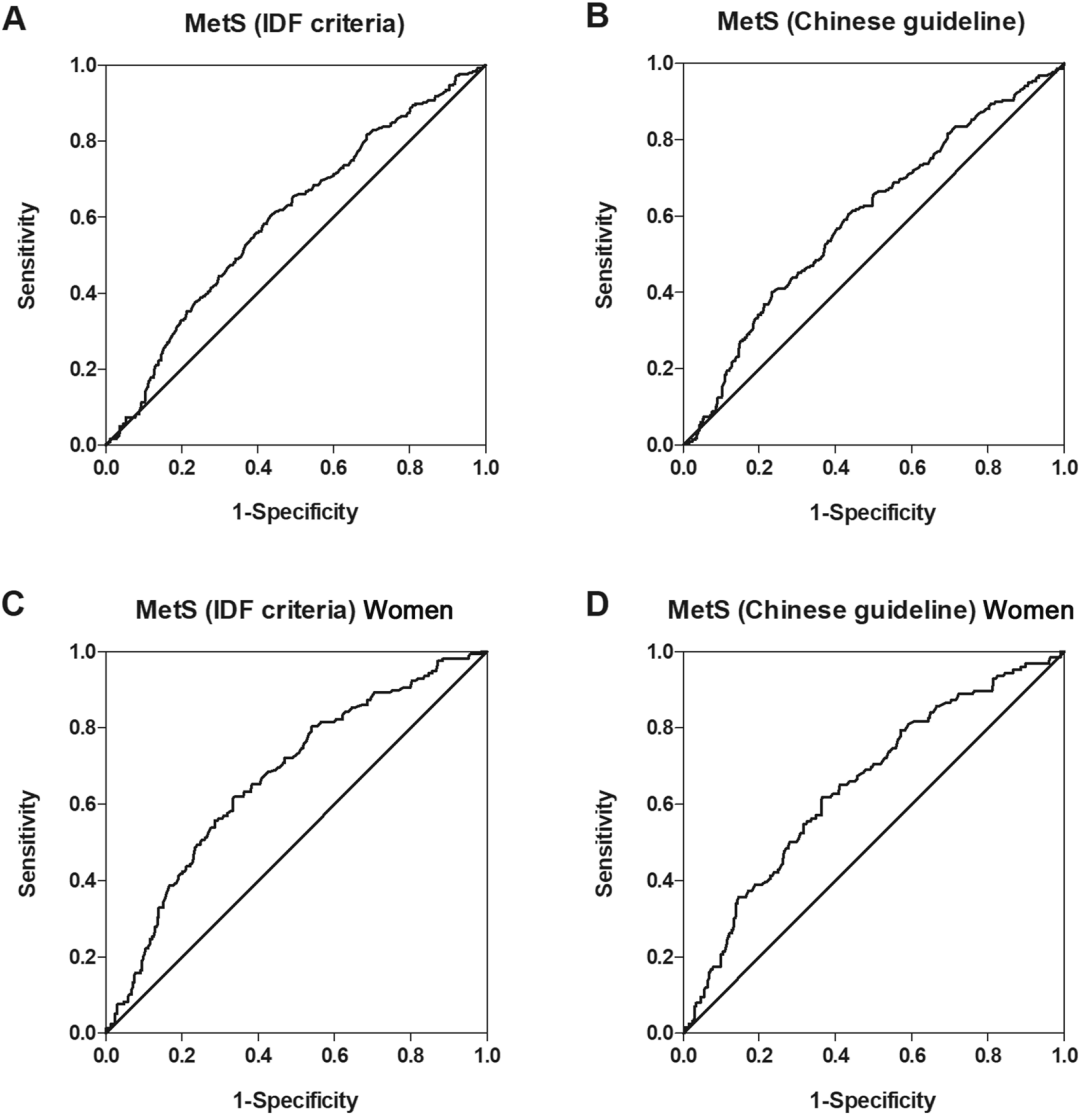
ROC curves of SUA level predicting MetS. (**A)** SUA level predicting MetS according to the IDF criteria in the very elderly. The AUC value was 0.622 (95%CI: 0.581–0.663, p < 0.001). (**B)** SUA level predicting MetS according to the Chinese guideline in the very elderly. The AUC value was 0.606 (95%CI: 0.563–0.649, p < 0.001). (**C)** SUA level predicting MetS according to the IDF criteria in very elderly women. The AUC value was 0.669 (95%CI: 0618–0.721, p < 0.001). (**D)** SUA level predicting MetS according to the Chinese guideline in very elderly women. The AUC value was 0.650 (95%CI: 0.594–0.706, p < 0.001). AUC: area under curve, CI: confidence interval, IDF: international diabetes federation, MetS: metabolic syndrome, ROC: receiver operating characteristic, SUA: serum uric acid.

The overall optimal SUA level cutoff point for predicting MetS (IDF criteria) was 351.5 µmol/L (Sensitivity: 60.3%, Specificity: 56.7%) in the elderly and 339.0 µmol/L (Sensitivity: 62.0%, Specificity: 66.3%) in very elderly women, respectively. The overall optimal SUA level cutoff point for predicting MetS (Chinese guideline) in the elderly was 352.5 µmol/L (Sensitivity: 60.4%, Specificity: 56.6%) and 339.0 µmol/L (Sensitivity: 61.9%, Specificity: 63.4%) in very elderly women, respectively (Fig. 1).

The area under curve (AUC) values for SUA level predicting MetS were 0.622 (95%CI: 0.581–0.663, p < 0.001) and 0.606 (95%CI: 0.563–0.649, p < 0.001) according to the IDF criteria and the Chinese guideline, respectively (Fig. 1). Likewise, the AUC values for SUA level predicting MetS in the very elderly women were 0.669 (95%CI: 0618–0.721, p < 0.001) and 0.650 (95%CI: 0.594–0.706, p < 0.001) according to the IDF criteria and the Chinese guideline, respectively (Fig. 1).

## Discussion

The main findings of this study are as follows: 1) MetS is common in the very elderly in Chengdu, especially in very elderly women. 2) hyperuricemia was generally associated with MetS in the very elderly in Southwest China.

### MetS in the very elderly

Because of the dramatic changes in the environment and rapid westernization of lifestyles such as more saturated fat and sugar consumption and less physical activity, the prevalence of MetS and ASCVD has been increasing rapidly during the past decades in China^2,3^. In addition, it has been shown that the prevalence of MetS is age-dependent. As per other studies in middle-aged and elderly Chinese ^14,20^, Korean^21^, and Japanese^22^, our findings show that the prevalence of MetS is remarkably high in the general community very elderly as well as in very elderly with hyperuricemia. The potential causes could be the clustering of more MetS components with aging in the very elderly (such as hypertension and abdominal obesity), decreased physical activities, and chilly and oil-rich local food style, which result in obesity and considerably dyslipidemia^23^.

Furthermore, following other studies^20,24^, this study also demonstrates that MetS is more common in very elderly women and those with hyperuricemia. Previous analysis has already showed a significantly higher prevalence of abdominal obesity and hypertriglyceridemia in very elderly women than it is in men^23^ and increased WC and extreme obesity increase more risk of MetS in women than in men^25^. Although the difference in the definition of MetS between the IDF criteria and the Chinese guideline results in the variation of the estimated prevalence of MetS without surprising, we could not ignore the fact that the prevalence of MetS is unexpectedly high in the very elderly residents in Chengdu, especially in very elderly women.

### Association between hyperuricemia and MetS

Elevated SUA level and hyperuricemia have already been reported to be associated with MetS and its components in middle-aged or elderly populations in other areas of China^14,15^. For the first time, this study describes the association between hyperuricemia and MetS in the community very elderly in Southwest China. The potential mechanisms for this association between hyperuricemia and MetS include inflammation and oxidative stress induced by SUA in adipocytes, which cause abnormal adipocytokine secretion and resulting in insulin resistance and the development of MetS^26,27^.

Previous studies^14,15,22,28^ establish that there is a gender difference in the prevalence of MetS. Furthermore, some studies^14,15,22,29^ show that the association between hyperuricemia and MetS is more evident in the middle-aged and elderly women. Following these findings, sub-analyses of our study find that there is a greater OR value of hyperuricemia for MetS in very elderly women than in men but without significant sex interaction. Therefore, whether hyperuricemia could differently contribute to the development of MetS in very elderly women and men, it still needs further prospective studies to clarify that.

In this very elderly population, hyperuricemia is found to be associated with abdominal obesity and dyslipidemia while not with hyperglycemia, which supports the results of some other studies^15,28^. One possible explanation may be that glucose can competitively inhibit SUA reabsorption and enhance its excretion at the same anatomical position in the gut and that SUA reduces after the onset of diabetes^30,31^.

It is well known that kidneys play a key role in SUA elimination, including glomerular filtration, tubular reabsorption, secretion and resorption^32^. However, renal function decreases with ageing even in apparently healthy individuals, but yet less in individuals with different chronic cardiovascular diseases^33^. Therefore, renal function can influence on the SUA level. Similar to the eGFR level in very elderly Chinese in CKD-EPI study^34^, the mean eGFR value in this study was around 60 ml/(min∙1.73m^2^), which might decrease SUA elimination and increase the SUA level. Taking this into consideration, it would not be surprising that the SUA level in this study is a little higher than that in the previous study with about 2000 Chinese elderly residents^35^.

The ability of SUA level for predicting a four year MetS incidence in general elderly Italian with an AUC value of 0.647 has been verified previously^29^. Similar to the results of that study, the AUC value in our research also indicates that the predictive ability of SUA for MetS is reasonable in the very elderly population in Chengdu.

In conclusion, the prevalence of MetS in the very elderly in Chengdu is relatively high and hyperuricemia is associated with the risk of MetS. On the other hand, it implicates that more efficient prevention strategies for cardiovascular and metabolic disease, including all MetS components and hyperuricemia, are urgently needed for middle-aged and elderly residents, especially for women^36^.

### Study limitations

Several limitations to be addressed in this study. First, this cross-sectional study could not reveal a causal relationship between hyperuricemia and MetS. Second, in this study, a two-hour postprandial glucose measurement was not performed, which might cause an underestimation of MetS. Third, the SUA level was a single measurement value, and previous dietary factors (i.e., drinks and meals) were not considered before measurement. Finally, our study could only describe epidemiological distribution in the very elderly in southwest China. Therefore, our findings may limit its generalizability to the very elderly population in other areas of China. Further prospective longitudinal studies are needed to investigate the possible role of SUA in MetS in the very elderly^36^.
